# Matrix metalloproteinases in subjects with type 1 diabetes

**DOI:** 10.1186/1472-6890-9-7

**Published:** 2009-09-16

**Authors:** Sedegheh Gharagozlian, Katja Svennevig, Hans-Jacob Bangstad, Jan-Olof Winberg, Svein Olav Kolset

**Affiliations:** 1 Department of Nutrition, Institute for Basic Medical Sciences, University of Oslo, Box 1046, Blindern, 0316 Oslo, Norway; 2 Department of Clinical Nutrition, Oslo University Hospital, Aker, Oslo, Norway; 3 Departments of Paediatrics, Woman and Child Division and Aker and Ullevål Diabetes Research Centre, Oslo University Hospital, Ullevål, 0407 Oslo, Norway; 4 Department of Medical Biochemistry, Institute of Medical Biology, University of Tromsø, 9037 Tromsø, Norway

## Abstract

**Background:**

Nephropathy is serious complication of diabetes. We have previously shown that level of the proteoglycan syndecan-1 in blood is associated with ultrastructural kidney changes in young persons with type 1 diabetes. Dysregulation of matrix metalloproteinases (MMPs) and tissue inhibitors of matrix metalloproteinases (TIMPs) may contribute to the development of nephropathy. The aim of this study was to investigate if the levels of MMPs in blood samples are potential markers of early nephropathy in type 1 diabetes.

**Methods:**

Blood samples were collected from type 1 diabetes patients after 11 years of diabetes (n = 15) and healthy volunteers (n = 12) and stored at ÷80°C until measurement. Levels and activities of serum MMP-2, MMP-9, TIMP-1 and TIMP- 2 were analyzed and compared to those of control individuals using ELISA, SDS-PAGE gelatin zymography, and Western blot analysis.

**Results:**

The serum levels of both MMP-9 and MMP-2 were significantly higher in subjects with type 1 diabetes, compared to controls (p = 0.016 and p = 0.008 respectively). Western blotting revealed no differences between the two groups in the levels of TIMP-1 or TIMP-2, respectively.

**Conclusion:**

Our MMP analysis of serum from a limited number of patients with type 1 diabetes suggest that such analysis is potentially useful as markers in studies of people at risk of progression to chronic kidney disease.

## Background

Diabetes mellitus (DM) represents a medical problem affecting millions of people world wide. Chronic hyperglycaemia, measured clinically as elevated glycosylated hemoglobin A1c (HbA_1c_), is the most important factor for the development and progression of microvascular complications like nephropathy, retinopathy and peripheral neuropathy in diabetes [1].

In the development of diabetic nephropathy, mesangial expansion and changes in the matrix of glomerular and tubular basement membranes are important aspects. The impact of long term hyperglycaemia on the development of structural changes (i.e. basement membrane thickening and mesangial expansion) in the kidney has been shown in studies of type 1 diabetes [2,3]. These changes can be arrested or reversed if the blood glucose level is improved [4] or normalized [5].

The extracellular matrix (ECM) in the basement membrane of the kidney glomeruli is of particular importance for the filtration properties. Structural changes in mesangial and basement matrix are related to proteinuria and hypertension and thus the progression of clinical diabetic nephropathy and kidney failure. One important class of molecules found in ECM and on cell surfaces and with functions in kidney filtration are the proteoglycans (PGs). We have recently shown that serum concentrations of the proteoglycan syndecan-1 is higher in subjects with type 1 diabetes and microalbuminuria than in those without microalbuminuria [6] suggesting that it is a potential serum marker for kidney changes.

Numerous classes of proteolytic enzymes probably participate in ECM degradation, and one class that appears to play a major role is MMPs [7] and their inhibitors, the TIMPs. MMPs have been shown to be increased in several diseases and secretion and activity to be strictly regulated. Gelatinase A (MMP-2) and gelatinase B (MMP-9) are the most important MMPs in normal kidneys and are therefore assumed to play major roles in basement membrane homeostasis [8].

Our studies on cultured human endothelial cells have established that primary human umbilical cord endothelial cells (HUVEC) exposed to hyperglycaemic conditions reduced secretion of MMP-2. MMP-9 secretion was negligible or very low in these cells, irrespective of treatment [9]. We have also established that HUVEC decreased the secretion of PGs including that of syndecan-1 under hyperglycemic conditions [10].

The aim of this study was to investigate if the activities and/or levels of MMPs in blood samples are markers of early nephropathy in type 1 diabetes

## Methods

### Patients

Blood samples were obtained from subjects with type 1 diabetes and microalbuminuria who participated in a prospective study. The study focused on blood glucose control and on morphological changes in the glomeruli. The inclusion criteria in this study were persistent microalbuminuria, defined as an AER between 15-200 μg/min in at least two out of three overnight urine samples taken during 1 year. At the time when the blood samples were obtained the mean duration of diabetes was 11.3 (7-18) and the mean age was 22 (19-30). The mean age of the controls was 31 (26-35) years. Details from this study have been presented [4]. In short, body mass index (BMI) was below 25 for all except one patient whose BMI was 29.6 (19.7-29.6). Further, only two patients were dyslipidemic with cholesterol/HDL cholesterol ratios of 6.9 and 9.5, respectively, mainly due to low HDL-cholesterol levels. The patients were all examined by the same investigator (HJB). Blood aliquots from 15 patients were taken and stored at -80°C. Healthy controls without type 1 diabetes (n = 12), male and female, were recruited from students and staff within the Department of Nutrition. These samples were also frozen. The present study focus on samples from the start of the study when the patients had microalbuminuria, but neither clinical nephropathy nor proliferative retinopathy, and all except one patient had blood pressure < 140/90 mmHg at the start of the study. All available samples were used. Samples were not subjected to thawing and freezing between sampling and analyses, to avoid loss of activity. All subjects in this study gave informed consent and the protocol was approved by the Regional Ethical Committee for Health Region South in Norway and conformed to the Helsinki Declaration.

### Zymography

SDS-PAGE gelatin-substrate zymography was used to analyze for gelatinases and urokinase. To detect gelatinases, SDS-PAGE gels contained 0.1% gelatin (Collagen type B from bovine skin, Sigma-Aldrich, Oslo, Norway). Positive control for proMMP-9 monomer (92 kDa) and homodimer (225 kDa) was conditioned serum-free medium from THP-1 cells. Positive control for active (62 kDa) and pro (72 kDa) forms of MMP-2 was conditioned serum-free medium from an osteosarcoma cell line.

The serum samples were mixed with sample buffer and loaded on 7.5% gels containing 0.1% gelatin. Protein concentrations of the samples were determined using the BC assay from Uptima (Interchim, Monttucon, France). After electrophoresis, the gels were incubated in 2.5% Triton X-100 to wash out SDS and then in assay buffer (0.05 M Tris-buffer pH 8.0 with 0.2 M NaCl, 0.005 M CaCl_2 _and 0.02% wt/vol Brij-35) overnight at 37°C to allow possible enzymes in the samples to degrade the gelatin matrix. In some experiments, wash and assay buffers contained the metalloproteinase inhibitor EDTA (10 mM).

To visualize gelatin lysis, gels were then stained with Coomassie blue and destained with ethanol: acetic acid: water (15: 5: 30). Areas containing gelatinolytic activity appeared as clear white zones on the blue-stained background. To obtain quantitative information the areas containing gelatinolytic activity were analysed in a Phosphoimager (Amersham Biosciences, UK).

### ELISA assay

The concentrations of MMP-2 and MMP-9 were determined in blood samples using ELISA kits from Amersham Biosciences, where the use of serum for these analyses was recommended.

### Western Blotting

Samples were mixed with Laemmli sample buffer, heated and electrophoresed in 10% (to detect MMP-9 and MMP-2) or 15% (to detect TIMP-1 and TIMP-2) SDS-PAGE gels (Bio-Rad, Hercules, CA, USA) and subjected to Western blotting as previously described [9].

### Statistical methods

Because variables were not normally distributed, nonparametric statistical analyses (Mann-Whitney tests) were used. Data are presented as means ± SEM or median values (interquartile intervals) as indicated. Comparison between groups of data was done by using box-plots. Statistical significance was defined as p < 0.05. All analyses were performed with SPSS for Windows version 16.

## Results

**Figure 1:**
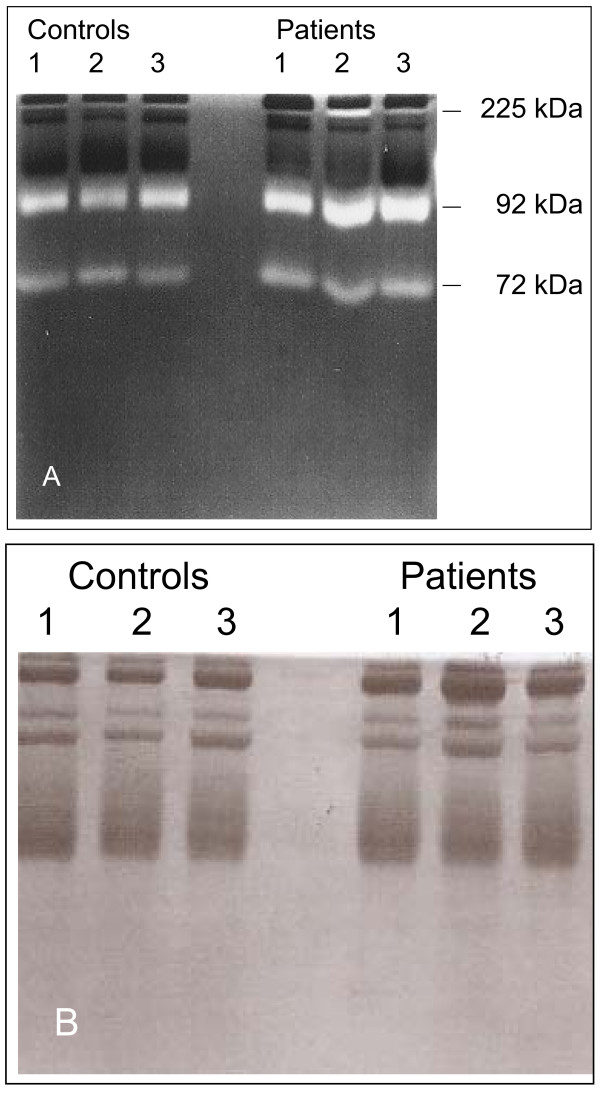
**SDS-PAGE zymography of serum samples**. Three serum samples taken from controls and three samples from subjects with type 1 diabetes were subjected to gel electrophoresis to detect possible gelatinolytic activities, in the absence (Panel A) and presence of 10 m EDTA (Panel B). The migration position of the 72 kDa (proMMP-2) and 92 kDa (proMMP-9) and dimeric proMMP-9 (225 kDa) standards are shown at the right side of panel A.

**Figure 2:**
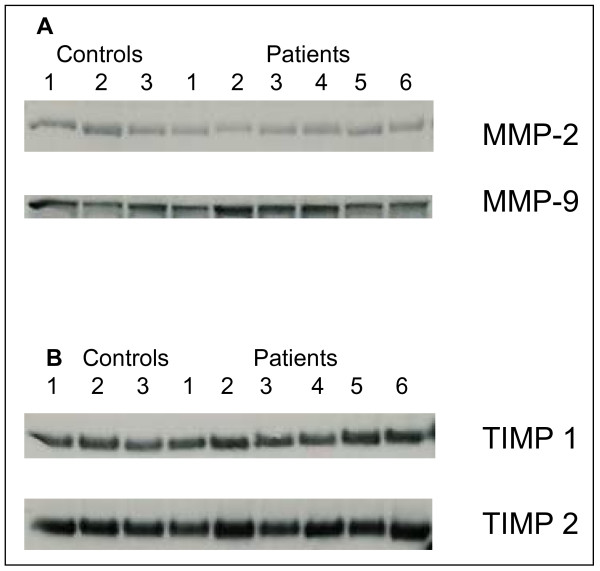
**Western blotting of serum samples**. Samples from subjects with type 1 diabetes and control samples were subjected to Western blotting using antibodies against MMP-2, MMP-9 (Panel A), and TIMP-1 and TIMP-2 (Panel B). Standards with MMP-2, MMP-9, TIMP-1 and TIMP-2 were subjected to the same procedures.

Blood samples from controls and subjects with type 1 diabetes were analyzed by SDS-PAGE gelatin zymography. In all blood samples, bands with gelatinase activities were detected at positions that corresponded with monomeric (92 kDa) and homodimeric (225 kDa) forms of proMMP-9, and proMMP-2 (72 kDa) in the standards (Figure 1A). These gelatinases did not appear when gels were washed and incubated in the presence of 10 mM EDTA (Figure 1B), which shows that they are metalloproteinases. The amounts of the 72 kDa gelatinase were consistently lower than those of the 92 kDa gelatinase in all experiments. To further identify the bands at 72 and 92 kDa, Western blot analysis was performed on reduced blood samples using antibodies against MMP-2 and MMP-9. This revealed that the 72 kDa band is proMMP-2 and the 92 kDa band is proMMP-9 (Figure 2A). During analysis we observed that almost all samples contained gelatinase activity in the top region of the gel. This most likely represents dimeric MMP-9, but has not been studied further.

**Figure 3:**
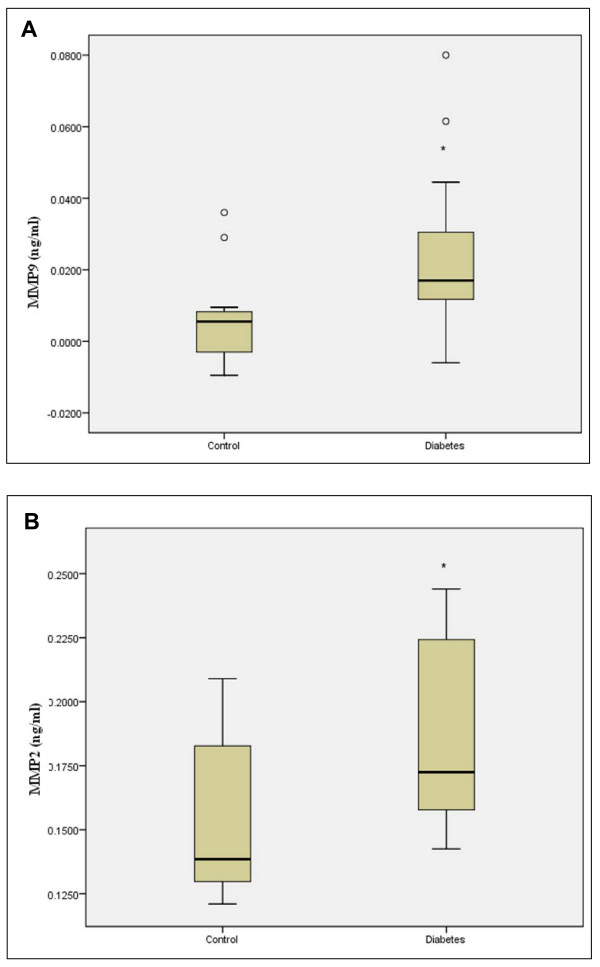
**Box plot analysis based on ELISA analyses**. The concentrations of MMP-9 (Panel A) and MMP-2 (Panel B) were determined in 12 control samples and 15 samples from persons with diabetes and these data were used for the box plot. In panel A there are two outliers in the control group. * p < 0.05.

We further determined the concentrations of MMP-2 and MMP-9 in samples from subjects with type 1 diabetes and controls using ELISA. As can be seen in Figure 3A the serum levels of MMP-9 were significantly higher in patients with diabetes (median: 0.0170 ng/ml; interquartile range, 0.0230 ng/ml) compared to healthy controls (median = 0.0055 ng/ml; interquartile range, 0.0145 ng/ml; p = 0.016). The levels of MMP-2 (Figure 3B) were also significantly higher in the diabetes group (median: 0.17250 ng/ml; interquartile range, 0.0735 ng/ml) compared to healthy controls (median = 0.13850 ng/ml; interquartile range, 0.0640 ng/ml; p = 0.008).

To investigate if TIMP concentrations differed in the obtained control and diabetes samples we performed Western blotting with antibodies against TIMP-1 and TIMP-2. Samples from both patients and controls contained detectable levels of TIMP-1 and TIMP-2. However, no apparent differences in the respective TIMPs could be observed in blood samples from the two different groups tested (Figure 2B).

## Discussion

In the present study serum from controls and from subjects with type 1 diabetes have been analysed for content of MMPs. The results presented show that the concentrations of MMP-9 and MMP-2 in serum were elevated in the patients. The results presented are from a limited number of patients with type 1 diabetes and conclusions should therefore be drawn with caution.

Results published on the use of MMPs as markers in relation to diabetic complications are somewhat conflicting. Increased MMP-2 levels, in particular in urine, have been related to increased risk of nephropathy [11]. Also, the use of MMP-9 as a surrogate marker for retinopathy has been suggested [12]. In addition, increased circulatory MMP-9, as well as MMP-2, has been reported in older subjects with type 1 diabetes [13]. In a study on children and adolescents, increased MMP-2 levels were suggested to be a marker of microangiopathy [14]. However, MMP-2 levels in plasma from subjects with diabetes type 1 and normal renal function did not differ from controls. Furthermore, MMP-9 was detected in patient plasma, but not in controls [15]. TIMP-1 concentrations were elevated in the diabetes group, but this change did not seem to be linked to vascular disease [15]. However, these studies were performed on plasma, whereas our studies were done on serum samples. These results referred to on MMP-2, but not those on MMP-9 and TIMP-1, support our findings.

Changes in the extracellular matrix are evident in the diabetic kidney [2,3]. It has been demonstrated that the thickness of the basement membrane is increased in subjects with type 1 diabetes, and that such changes can be arrested by improved blood glucose control [4]. The changes in the ECM of the kidney (i.e. glomeruli and tubuli) contribute to the development of albuminuria, and at the end kidney failure. The question is if the blood concentrations of MMPs, TIMPs and PGs are potential markers for such changes. Sensitive markers can increase our understanding of the process leading to kidney failure and possibly detect subjects with high risk of progression to chronic kidney disease which again would facilitate early treatment.

In a previous study syndecan-1 was shown to be elevated in blood samples from the same subjects studied here, who all had microalbuminuria [6]. We did not find any correlation between elevated syndecan-1 concentrations and neither MMP-9 nor MMP-2 levels in these samples (unpublished observation). Although our results here show elevated levels of MMP-9 and MMP-2 it is tempting to speculate that syndecan-1 levels in blood may be a better marker for kidney changes. In future studies it would be of interest to compare MMP levels with syndecan-1 levels in larger cohorts of persons with type 1 or type 2, comparing those with and without microalbuminuria.

## Conclusion

Our MMP analysis of serum from a limited number of patients with type 1 diabetes suggest that such analysis is potentially useful as markers in studies of people at risk of progression to chronic kidney disease.

## Competing interests

The authors declare that they have no competing interests.

## Authors' contributions

HJB and SOK are responsible for conceiving and designing the study. HJB collected the blood samples and SG, KS and JOW acquired the data. SG made the figures and performed the statistical analyses. SOK drafted the manuscript which was revised by all authors.

## Pre-publication history

The pre-publication history for this paper can be accessed here:


